# The Effect of Normobaric Hypoxic Confinement on Metabolism, Gut Hormones, and Body Composition

**DOI:** 10.3389/fphys.2016.00202

**Published:** 2016-06-02

**Authors:** Igor B. Mekjavic, Mojca Amon, Roger Kölegård, Stylianos N. Kounalakis, Liz Simpson, Ola Eiken, Michail E. Keramidas, Ian A. Macdonald

**Affiliations:** ^1^Department of Automation, Biocybernetics and Robotics, Jožef Stefan InstituteLjubljana, Slovenia; ^2^Department of Biomedical Physiology and Kinesiology, Simon Fraser UniversityBurnaby, BC, Canada; ^3^Jožef Stefan International Postgraduate SchoolLjubljana, Slovenia; ^4^Department of Environmental Physiology, Swedish Aerospace Physiology Center, School of Technology and Health, Royal Institute of TechnologyStockholm, Sweden; ^5^Human Performance-Rehabilitation Laboratory, Faculty of Physical and Cultural Education, Hellenic Military UniversityVari, Greece; ^6^Metabolic Physiology Group, Faculty of Medicine and Health Sciences, University of Nottingham Queen's Medical CentreNottingham, UK

**Keywords:** altitude, hypoxia, metabolism, appetite, energy expenditure

## Abstract

To assess the effect of normobaric hypoxia on metabolism, gut hormones, and body composition, 11 normal weight, aerobically trained (O_2peak_: 60.6 ± 9.5 ml·kg^−1^·min^−1^) men (73.0 ± 7.7 kg; 23.7 ± 4.0 years, BMI 22.2 ± 2.4 kg·m^−2^) were confined to a normobaric (altitude ≃ 940 m) normoxic (NORMOXIA; P_I_O_2_ ≃ 133.2 mmHg) or normobaric hypoxic (HYPOXIA; P_I_O was reduced from 105.6 to 97.7 mmHg over 10 days) environment for 10 days in a randomized cross-over design. The wash-out period between confinements was 3 weeks. During each 10-day period, subjects avoided strenuous physical activity and were under continuous nutritional control. Before, and at the end of each exposure, subjects completed a meal tolerance test (MTT), during which blood glucose, insulin, GLP-1, ghrelin, peptide-YY, adrenaline, noradrenaline, leptin, and gastro-intestinal blood flow and appetite sensations were measured. There was no significant change in body weight in either of the confinements (NORMOXIA: −0.7 ± 0.2 kg; HYPOXIA: −0.9 ± 0.2 kg), but a significant increase in fat mass in NORMOXIA (0.23 ± 0.45 kg), but not in HYPOXIA (0.08 ± 0.08 kg). HYPOXIA confinement increased fasting noradrenaline and decreased energy intake, the latter most likely associated with increased fasting leptin. The majority of all other measured variables/responses were similar in NORMOXIA and HYPOXIA. To conclude, normobaric hypoxic confinement without exercise training results in negative energy balance due to primarily reduced energy intake.

## Introduction

A common consequence of exposure to high altitude (>2500 m) is an unexplained loss of body weight (Boyer and Blume, 1984; Guilland and Klepping, 1985; Rose et al., 1988; Westerterp et al., 1992; Pulfrey and Jones, 1996; Barry and Pollard, 2003), which appears to be dependent on the nature of the hypoxic stimulus (Kayser, 1994; Mazzeo, 2008). Weight loss at high altitude was observed even in the absence of symptoms of mountain sickness (Tschöp and Morrison, 2001), which is known to affect appetite. Most of the field studies conducted to date have not been able to control for potentially confounding variables, such as cold, physical exertion, limited palatable food at altitude, thus the effect of hypoxia *per se* on the etiology of “high altitude anorexia” remains unresolved.

The suggested likely reasons for body-weight loss at altitude are a decrease in appetite, and the associated decrease in energy intake (Kalson et al., 2010). Laboratory trials have demonstrated appetite suppression after a simulated 31-day hypobaric hypoxic exposure to 8848 m (Westerterp-Plantenga et al., 1999), and it was suggested that the hormone leptin may mediate altitude anorexia (Tschöp et al., 1998). Leptin serves as an adiposity signal to inform the brain of the adipose tissue mass in a negative feedback loop regulating food intake and energy expenditure. However, observations of the effect of hypoxia on leptin levels are conflicting. Namely, studies have reported increased (Shukla et al., 2005; Snyder et al., 2008), decreased (Zaccaria et al., 2004; Vats et al., 2007), or unchanged (Barnholt et al., 2006) leptin levels during hypoxic exposure. Moreover, the endocrine factors that contribute to the observed changes in body weight at altitude remain unclear. It has been suggested that absorption of nutrients is slowed, and possibly impaired, at altitude (Milledge, 1972; Schoots et al., 2006). Recently, the observation of suppression of energy intake and of the appetite hormone, ghrelin, after a 7-h normobaric hypoxic exposure, suggested that short-term hypoxia *per se*, in the absence of cold and other stressors may be implicated in high altitude anorexia (Wasse et al., 2012). However, after prolonged hypoxia (range of duration: 7 days to 7 weeks), ghrelin was unchanged at altitude (Shukla et al., 2005). Shukla et al. (2005) reported that the peptide YY (PYY), which reduces appetite or promotes satiety, was not significantly altered after acute normobaric hypoxia. Thus, the effect of prolonged (a few days) normobaric hypoxia on this gut hormone, which could potentially contribute to weight loss, remains unresolved.

Energy expenditure (EE) is influenced by oxygen supply. However, the observations of the effect of hypoxia on resting EE (REE) are conflicting. Though the majority of studies report an increase (Picon-Reategui, 1961; Grover, 1963; Gill and Pugh, 1964; Butterfield et al., 1992), a few have shown no change in altitude REE (Durnin and Brockway, 1959; Westerterp et al., 1992, 1994). Of the former, values of EE in climbers have been reported to be comparable to highly trained endurance athletes at sea level (Westerterp et al., 1992).

Weight loss resulting from an imbalance between energy intake and expenditure may be related to other factors, such as nutrient intake, digestibility and altered fluid balance. Weight loss at altitude has also been attributed to the initial loss of water, and subsequent loss of fat and muscle mass due to malnutrition (Kayser, 1994). Westerterp (2001) concluded that weight loss is mainly caused by malnutrition due to hypoxia-related satiety, independent of acute mountain sickness. Moreover, decreased xylose absorption with more severe desaturation was noted (Milledge, 1972), confirming the notion that hypoxia followed by reoxygenation impairs nutrient absorption (Schoots et al., 2006). However, hypoxia is believed to be a major factor in the development, and progression of metabolic alterations in glucose metabolism (Stock et al., 1978; Louis and Punjabi, 2009). The observed reduction in baseline and postprandial gut blood flow [superior mesenteric artery (SMA)] during acute altitude exposure has been attributed to increased intestinal sympathetic tone, and it has been suggested that this could lead to reduced energy intake during prolonged exposure (Loshbaugh et al., 2006).

The above empirical, and also anecdotal evidence of weight loss following high altitude expeditions, suggests that hypoxia may also induce weight loss in athletes conducting hypoxic training (i.e., simulated altitude training) to improve performance (Richalet and Gore, 2008; Siebenmann et al., 2012). Such weight loss may be beneficial, if it affected the fat tissue compartment, but detrimental if it reduced muscle mass, and/or reflected dehydration. Without knowledge of the nature of hypoxia-induced reductions in body mass, advocating hypoxic training in weight-loss programmes may be unwarranted (Netzer et al., 2008; Wasse et al., 2012). As a prelude to a study investigating the effect of normobaric hypoxia on the metabolism in overweight and obese individuals, we initiated the present study on normal weight and active subjects. Consequently, this study exposed individuals to a moderate altitude of only 3000 m, mainly due to precautionary measures, and partly since the majority of the studies to date have been conducted at higher altitudes. A principal concern in establishing the protocol of this and future such studies, was that hypoxia may initiate a significant cardiorespiratory stress, which may be acceptable in normal weight active subjects, but perhaps of more concern in an overweight, inactive subject population. Since very few studies have been conducted with the latter population at altitude, a conservative approach to hypoxic exposure of such individuals was warranted. An additional concern was that the obstructive sleep apnea observed in overweight and obese individuals might be augmented by hypoxia-induced central sleep apnea, and thus might pose an additional cardiorespiratory stress. The present study was therefore not designed to determine the threshold altitude at which changes in metabolism would be observed, but to determine the changes in metabolism, if any, at an altitude that would be considered safe for overweight and obese individuals, should the responses observed in normal weight subjects warrant consideration of hypoxia as a potential factor for inducing weight reduction.

The present study tested the hypothesis that a continuous 10-day hypoxic confinement in healthy normal weight males induces metabolic changes reflected in changes in REE, gastrointestinal hormones and appetite, which may lead to loss of body weight, and in turn may provide some insight into the phenomenon of high-altitude anorexia.

## Methods

### Subjects

Eleven healthy aerobically trained males (mean ± SD age and stature of 23.7 ± 4.0 years and 181 ± 9 cm), all low altitude (351 ± 103 m; F_I_O_2_ = 0.21) residents, participated in two 10-day confinements. Subject exclusion criteria included a history of physician-diagnosed medical problems, recent prolonged exposure to normobaric or hypobaric hypoxia, or recent weight loss, participation in any dietary manipulations in the past 6 months, and use of any medications or drugs. All participants gave their written informed consent to participate in the study. The study protocol was approved by the National Committee for Medical Ethics at the Ministry of Health of the Republic Slovenia and conformed to the Declaration of Helsinki.

### Experimental protocol

**Figure 1:**
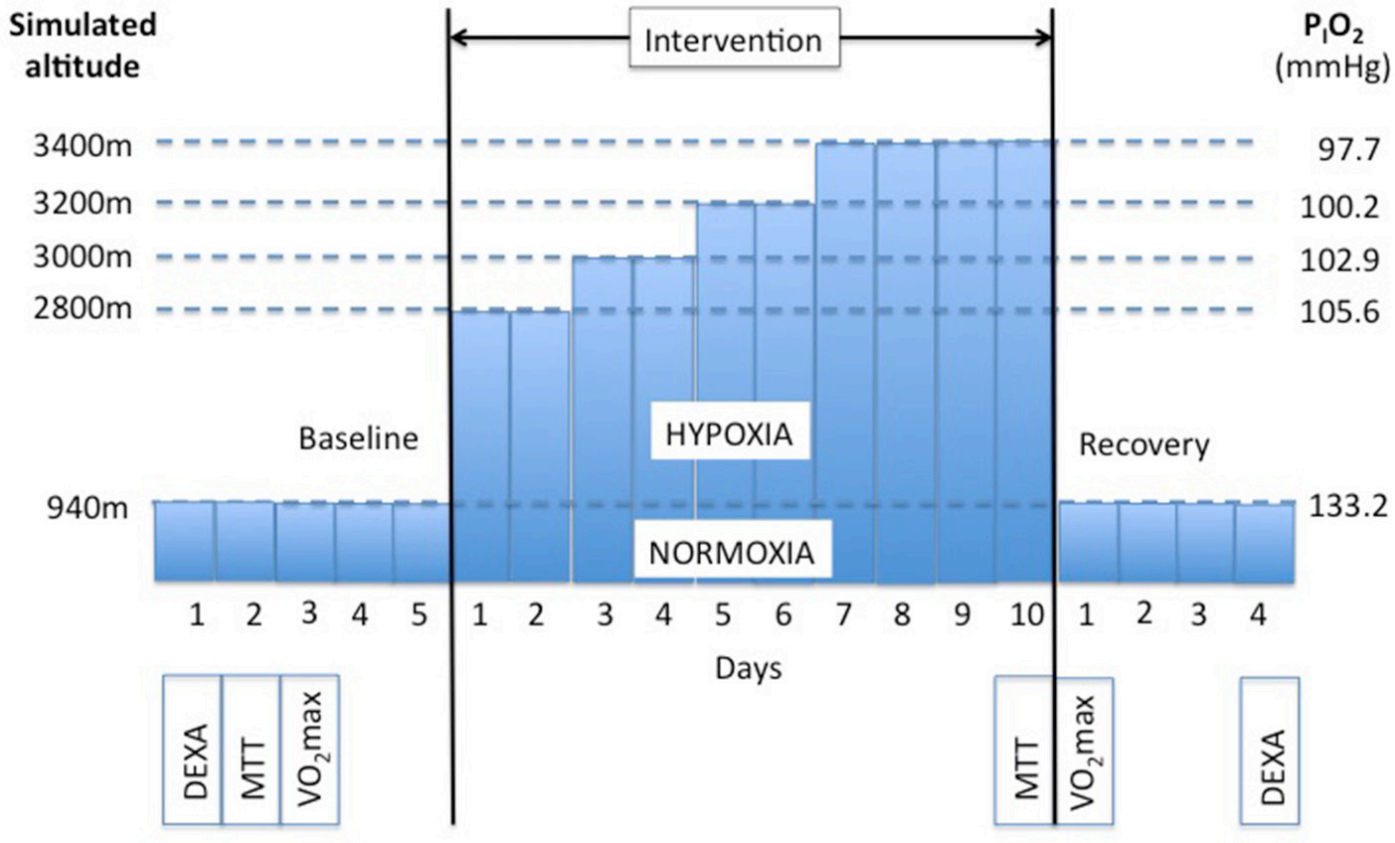
**Experimental protocol**. Subjects (*N* = 11) participated in two 10-day confinements. In one they were confined to a normobaric normoxic environment (NORMOXIA), and in the other to a progressive normobaric hypoxic environment (HYPOXIA). Each confinement was preceded by a 5-day period, during which baseline measurements were obtained (DEXA, Body composition was measured with Dual Energy X-Ray Absorption; MTT, Meal Toelrance Test; VO_2_max, maximal oxygen uptake test). Subjects remained at the facility 4 days following each confinement for post measurements.

The study was conducted at the Olympic Sport Centre Planica (Rateče, Slovenia) situated at an altitude of 940 m. Subjects participated in two confinements, during which they were confined to one floor of the facility. The study was designed as a randomized cross-over study. Participants were assigned to two groups: one group (*n* = 6) was initially confined to a normobaric normoxic environment (NORMOXIA; inspired fraction of O_2_, F_I_O_2_ = 0.209; P_I_O_2_ ≃ 133.2 mmHg) for 10 days and the other (*n* = 5) to a controlled normobaric hypoxic environment (HYPOXIA). As shown in Figure 1, subjects were exposed to a simulated altitude of 2800 m (P_I_O_2_ = 105.6 mmHg) for the first 2 days (days 1 and 2). Thereafter the fraction of oxygen in the facility was reduced every 2 days, so that the subjects were exposed to a simulated altitude of 3000 m (P_I_O_2_ = 102.9 mmHg) on days 3 and 4, to 3200 m (P_I_O_2_ = 100.2 mmHg) on days 5 and 6, and finally to 3400 m (P_I_O_2_ = 97.7 mmHg) for the remaining 4 days. In both trials, the participants arrived at the facility 5 days prior to the onset of the 10-day confinement. During these 5 days, they were familiarized with the experimental protocol and equipment, and baseline measurements were obtained. Upon completion of the 10-day confinements, subjects remained at the facility for an additional 4 days for the post-confinement tests. The meal tolerance test (MTT) and the assessment of resting oxygen uptake ($\overset{\cdot }{\text{V}}$O_2_rest) were performed before and after each confinement. In the HYPOXIA confinement, once these tests were completed, the subjects remained under normoxic conditions for the next 4 days. After a 3-week wash-out period the subjects returned to the Olympic Sport Centre Planica, and the conditions were crossed-over.

During both the NORMOXIA and HYPOXIA trials, subjects were confined to the living quarters (each group had at their disposal 3 double sleeping rooms and one living/dining room; total area of ~90 m^2^). In the HYPOXIA trial, the reduction in F_I_O_2_ was achieved with an oxygen dilution system (B-cat, the Netherlands), based on the Vacuum-Pressure Swing Adsorption principle. The oxygen levels in each room were monitored and recorded at 15-min intervals throughout the 10-day period. In the event that the oxygen fraction in any given room decreased below the pre-set value, delivery of the hypoxic gas mixture to that room was stopped. In the event that the oxygen fraction dropped by more than 0.5% of the pre-set value, the control system activated a fan, which delivered external ambient air into that room. As a consequence of the fan being activated, the oxygen fraction in the room would increase rapidly to the desired level. Once the oxygen fraction attained the pre-set value, the fan would be de-activated. Each subject was also requested to either wear, or have in close proximity, a personal clip-on type of environmental oxygen analyser (Rae PGM-1100, California, USA) with an audible alarm that was activated in the event that the oxygen level decreased below the pre-set level.

Prior to the onset of the study, subjects completed questionnaires regarding their current and past health status, habitual physical activity levels, dietary habits, and food preferences. During the 5-day period prior to each confinement, subjects were requested to record their dietary intake in a food diary. During the 10-day confinement period, subjects were restricted from any high-intensity, or aerobic exercise. Physical activity was restricted to slow walks in the living area. Nutritional choices and energy intake were documented in daily nutritional diaries during both trials. The food menu comprised typical national foods, freely available, and an effort was made to accommodate individual food preferences. The subjects received the same food menu during each 10-day exposure, but without any restriction regarding the quantity consumed; they ate and drank *ad libitum*. The daily energy intake was recorded and analyzed with a dietary assessment program (OPKP, Jozef Stefan Institute, Ljubljana, Slovenia). Subjects' well-being was monitored by medical personnel. All participants had personal pulse oxymeters (Nonin, Medicals 3100 WristOx, Minnesota, USA) monitoring capillary oxyhemoglobin saturation (SpO_2_) and heart rate (HR). Symptoms of mountain sickness and individual mood and appetite were monitored daily with the Lake Louise Scoring system (LLS) and Visual Analog Scale (VAS) for Mood and Appetite, respectively. The metabolic tests were conducted in a normoxic environment, 3 days prior to the onset of each 10-day confinement (pre-test), and on the morning of the completion of the confinement period (post-test). Namely, the confinements commenced at 9:00 a.m. on day 1, and were terminated at 9:00 on day 11.

### Diet

The menus were constructed using the application Open Platform for Clinical Nutrition (OPKP; www.opkp.si) developed at the Jozef Stefan Institute (Ljubljana, Slovenia). Five daily meals (breakfast, morning snack, lunch, afternoon snack, dinner) were prepared by the kitchen staff of the Olympic Sport Centre Planica, and the components weighed to ±1 g accuracy. Meals were served at the same time of day during both normoxic and hypoxic trials. The analysis of energy, macronutrient, and fluid intake was conducted for each subject using the OPKP programme. An example of a 10-day menu has been presented previously (Debevec et al., 2014).

### Anthropometry

Body composition was analyzed before and after each 10-day confinement with Dual-Emission X-ray absorptiometry (Discovery, Hologic, Inc., Bedford USA). Measurements were made of total body fat mass and regional fat mass (abdominal, right thigh, left thigh) and fat-free mass. Body weight and height were measured with a weighing scale and a stadiometer, respectively (Seca 703, Seca, Hamburg, Germany).

### Aerobic capacity

All subjects performed an incremental exercise test to exhaustion on a cycle-ergometer (Daum electronics, Fürth, Germany) 3 days prior to, and 1 day after, each confinement (Figure 1), to determine their $\overset{\cdot }{\text{V}}$O_2peak_. The trial was performed around noon. The post-trial was carried out ~3 h after the termination of the confinement; during this period, subjects, who were always supervised by an assistant, were requested to stay in the facility, and not to engage in any strenuous physical activity. Also, according to the schedule, subjects consumed a standardized breakfast around 7:30 a.m., and a light snack (i.e., fruit) approximately an hour before the trial. The $\overset{\cdot }{\text{V}}$O_2_peak testing protocol comprised a 5-min resting period, during which subjects remained still and seated on the cycle-ergometer and $\overset{\cdot }{\text{V}}$O_2_rest was determined.

After a 2-min warm-up at 60 W, the workload was incremented each minute by 25 W, until exhaustion. Inability to maintain the cycling cadence above 60 rpm, plateau in $\overset{\cdot }{\text{V}}$O_2_ and/or a respiratory exchange ratio >1.1, were the criteria used, to confirm the attainment of the $\overset{\cdot }{\text{V}}$O_2peak_ (calculated as the highest VO_2peak_ averaged over 60 s during the test). $\overset{\cdot }{\text{V}}$O_2_ was measured breath-by-breath with a Quark CPET metabolic cart (Cosmed, Quark PFT, Rome, Italy).

### Metabolic test

Prior to, and on the final day of each 10-day confinement, subjects completed a MTT to assess their fasting metabolic status and the postprandial metabolic responses. For 2 days before the pre-confinement metabolic experiments, subjects refrained from exercising and were under dietary control with limited caffeine (daily maximum caffeine content: 150 mg) and alcohol (daily maximum alcohol content: 8 g or 10 ml) consumption. The dietary intake diaries obtained prior to each MTT revealed that the macronutrient composition was similar for the NORMOXIA and HYPOXIA trials. The last meal prior to the MTT was the evening meal the day before the test. For each subject and MTT, the composition of the last meal prior to the 12-h fast was the same.

One hour after subjects completed their morning personal hygiene and morning body weighing, they relaxed and rested supine for 3 h during the metabolic test. The metabolic tests were conducted at the same time of the day for each subject (between 07:00 a.m. and 01:00 p.m.); the environmental conditions were kept constant: the mean ambient temperature and relative humidity were 25 ± 0.8°C and 37.5 ± 4.7%, respectively.

For the MTT, subjects consumed a standardized mixed nutrient liquid test meal (Ensure, Nutrition shake, vanilla flavor; Abbott) based on their individual body weight (5 ml·kg^−1^, 1.5 kcal·ml^−1^) determined on the day of the experiment. Additionally, ^13^C-labeled glucose (9.2 mg·kg^−1^) was added to the test meal as an indicator of gastric emptying, and glucose uptake and use (expired ^13^CO_2_ was measured). The average meal volume was 361.4 ± 3.4 ml and contained 27.4 g of protein, 48.5 g of fat, and 88.4 g of carbohydrate, plus significant amounts of vitamins and minerals. Participants were asked to consume the entire meal portion provided.

### Blood sampling

Blood samples (6 ml) were drawn at regular intervals through a catheter (Baxter Health Care, Valencia, CA) inserted into a dorsal hand vein at the beginning of the experiment. The subject's catheterised hand was placed in a hot air box (50–55°C) to maintain a constant high hand skin temperature and provide arterialised venous blood. Arterialised venous blood samples were collected in the fasted state prior to the test meal (one sample 15 min before, and another ~10 s before the meal), and at regular intervals during the 2 h following the ingestion of the test meal, to monitor blood glucose (every 10 min), and to analyse the responses of serum insulin and plasma glucagon-like peptide (GLP-1), peptide YY (PYY), and ghrelin (every 15 min). Plasma catecholamine (noradrenaline and adrenaline) responses were additionally measured in the fasting state and post-prandially. The plasma level of leptin was also measured in the fasted state before and after each confinement.

The blood glucose level was analyzed immediately from each arterialised venous blood sample (HemoCue, HMC-201-PROMO, Sweden). For subsequent hormone analyses, all blood samples were either immediately (or after 10 min, in the case of the insulin tube to allow clotting) centrifuged at 3000 rpm for 10 min (at a temperature of 4°C), and aliquots kept on ice until the end of the MTT and stored at −20°C for 1 day, and thereafter at −70°C for further analyses. The analysis of all plasma samples was conducted in duplicate using the assays described below.

Insulin concentrations were determined using radio-immunoassay (RIA) (Insulin Coat-A-Count, Diagnostic Products Corp., Los Angeles, USA); GLP-1 concentrations (comprising GLP-1 7–36 amide and 7–37) using Enzyme-Linked ImmunoSorbent Assay (ELISA) (Merck Millipore, Missouri, USA); PYY using RIA for total PYY (Merck Millipore, Missouri, USA), which recognized both PYY1-36 and PYY3-36 in EDTA plasma plus aprotinin; ghrelin was determined using RIA for total ghrelin (Merck Millipore, Missouri, USA) and leptin was determined using a human leptin RIA (Merck Millipore, Missouri, USA). Catecholamines were measured using extraction of the adrenaline and noradrenaline from the plasma (which had been treated with EGTA/glutathione (Sigma-Aldrich, Dorset, UK) as a preservative) followed by high performance liquid chromatography (HPLC) with electrochemical detection (Forster and MacDonald, 1998).

Labeled glucose artificially enriched with (U-^13^C) glucose (^13^C/C >99%; Isotec, Miamisburg, OH, USA) was dissolved in each individual's liquid meal so that the appearance of ^13^CO_2_ in the expired air could be used as a marker of the combination of gastric emptying, uptake and oxidation of the test meal. This breath testing is based on the principle that an ingested substrate is metabolized, and a measurable metabolite is then expelled by the respiratory system. Therefore, breath samples were collected simultaneously with the blood samples before and after the meal (at −15, 0, 15, 30, 45, 60, 75, 90, 105 and 120 min) using a breath-sampling bag (500 ml) with a one-way valve for capture of normal exhaled air. To allow analysis of ^13^C/^12^C in expired CO_2_, 20 ml samples of expired gases were collected from the breath-sampling bag via a catheter (Baxter Health Care, Valencia, CA) into evacuated tubes (vacutainers: Becton Dickinson, Franklin Lakes, NJ) and stored until further analysis. All samples were subsequently analyzed in triplicate with mass spectrometry (Prism, VG, Manchester, UK) and ^13^C abundance was calculated for all time points. The incremental postprandial response (i.e., the ^13^C enrichment curve) was integrated and presented as an area under the curve (AUC).

### Appetite sensation

Subjective perceptions of hunger, fullness, desire to eat, thirst and prospective food consumption were assessed using a validated visual analog scale (VAS; Stubbs et al., 2000). The VAS is a 100 mm line, anchored to the left with “sensation not felt at all” and to the right with “sensation felt the greatest”; subjects were asked to place a vertical line in relation to their feeling at that particular point in time, these scores were then summed to form the Composite Satiety Score (CSS) according to the following equation (the higher the score, the higher the level of subjective satiety).

CSS=(Full + (100 − Desire) + (100 − Hunger) + (100 − PFC))/4

### Intestinal blood flow

Blood flow was measured in the proximal portion of the SMA, 2–5 cm distally to its origin in the aorta. Flow was estimated by measurements of vessel-lumen diameter and mean flow-velocity, using ultrasonographic/Doppler techniques (MicroMaxx, SonoSite Inc., WA, USA). Flow velocity was measured using a 2–5 MHz linear array Doppler transducer that was kept aligned with the vessel; the angle correction of the transducer was maintained at < 60° and its sample volume was adjusted to cover more than 75% of the vessel lumen. The diameter of the SMA was measured in B-mode image during end-diastole (determined from the ECG), as wall-to-wall distance in the sagittal section. Assuming that the artery had a circular cross-section, flow was subsequently calculated by multiplying vessel cross-sectional area by the mean flow-velocity. Each flow determination was the average of measurements from 3 to 4 consecutive heart beats, repeated 2–3 times, i.e., the average of data from 6 to 12 beats. The same sonographer performed all measurements.

### Calculations and statistical methods

Mass spectrometer analysis of breath samples provided a delta value (δ), which was used to calculate abundance (atom %) at each time point using standard methods (Pouteau et al., 1998). Enrichment of breath samples was then obtained by standardizing postprandial values to those at baseline.

The homeostatic model assessment (HOMA) method was used to quantify fasting insulin resistance and β-cell function (Matthews et al., 1985). The nonlinear model of two types of HOMA scores were used:

$$\begin{array}{l} \text{HOMA IR}=\text{insulin resistance }=\\ \;=(\text{fasting insulin in mU}\cdot {\text{l}}^{-1})\\ \;\times (\text{fasting plasma glucose in mmol}\cdot {\text{l}}^{-1})/22.5\\ \text{HOMA β}=\text{β-cell function}[\%]\;=\\ \;=20\times (\text{fasting insulin in mU}\cdot {\text{l}}^{-1})/\\ \;((\text{fasting glucose in mmol}\cdot {\text{l}}^{-1})-3.5). \end{array}$$

A 2-way ANOVA (NORMOXIA-HYPOXIA, Pre-Post) with repeated measures was used to define the effect of the 10-day confinements on the measured variables. A Tukey *post hoc* test was used to assign the specific differences in the analysis of variance. Additionally, some data were presented as calculated area under curve (AUC) and compared with the same type of ANOVA. Differences between the post HYPOXIA and post NORMOXIA confinement cardiorespiratory responses and haematological values were compared with a one-tailed *t*-test. Values are mean ± SD unless indicated otherwise. The significance level was set at 0.05.

## Results

There was no significant change in body mass in the NORMOXIA, and HYPOXIA confinements. Total body fat mass significantly increased in NORMOXIA (*p* = 0.04), whereas no difference between pre- and post-measurements was observed in HYPOXIA (Table 1). In the abdominal region, absolute fat content increased in NORMOXIA (*p* = 0.01), but exhibited a tendency toward a decrease in HYPOXIA (*p* = 0.08). There was also a tendency, albeit not significant, of a reduction in lean body mass after NORMOXIA (*p* = 0.06).

**Table 1:** **Body composition, cardiorespiratory and hematological responses before (PRE) and after (POST) the 10-day NORMOXIA and HYPOXIA confinements**.

**Variable**	**NORMOXIA**		**HYPOXIA**	
	**Pre**	**Post**	**Pre**	**Post**
**BODY COMPOSITION**				
Body mass (kg)	73.0 ± 7.7	72.4 ± 7.0	72.3 ± 5.9	71.4 ± 6.3
Body mass index (BMI, kg·m^−2^)	22.3 ± 2.4	22.0 ± 2.7	22.1 ± 1.8	21.8 ± 2.0
Total fat mass (%)	14.6 ± 5.8	15.0 ± 6.1	14.6 ± 6.1	14.6 ± 5.5
Total fat mass (kg)	10.9 ± 5.5	11.1 ± 5.4^*^	10.7 ± 5.3	10.6 ± 4.8
Abdominal fat mass (%)	11.9 ± 6.3	12.7 ± 6.7^*^	11.9 ± 6.6	11.8 ± 5.8
Right thigh (%)	14.9 ± 6.9	15.9 ± 7.3	14.9 ± 6.6	14.5 ± 6.7
Left thigh (%)	15.3 ± 6.3	15.5 ± 6.2	14.9 ± 6.5	14.9 ± 6.0
Fat free mass (%)	85.4 ± 5.8	85.0 ± 6.0^*^	85.4 ± 6.1	85.4 ± 5.5
Fat free mass (kg)	62.2 ± 5.2	61.3 ± 5.0	61.6 ± 4.9	60.8 ± 4.7
**RESTING CARDIORESPIRATORY RESPONSES**				
Heart rate (beats·min^−1^)	77 ± 13	81 ± 15	79 ± 16	85 ± 13
$\overset{\cdot }{\text{V}}$O_2_ (ml·kg^−1^·min^−1^)	7.33 + 1.03	6.86 + 1.12	7.29 + 1.09	7.36 + 1.26^†^
$\overset{\cdot }{\text{V}}$_E_rest (L. min^−1^)	15.4 + 2.09	14.21 + 2.06	14.73 + 2.05	15.89 + 1.47^†^
RQ	0.84 + 0.05	0.86 + 0.04	0.87 + 0.1	0.84 + 0.05^†^
**MAXIMUM OXYGEN UPTAKE**				
$\overset{\cdot }{\text{V}}$O_2_ (ml·kg^−1^·min^−1^)	61.2 ± 7.2	61.8 ± 7.0	62.6 ± 6.1	62.4 ± 5.9
**HEMATOLOGICAL VARIABLES**				
Hemoglobin (g·l^−1^)	146.4 ± 0.9	144.5 ± 0.8	147.2 ± 0.9	148.4 ± 0.9^†^
Hematocrit (%)	44.2 ± 2.4	43.9 ± 2.3	44.9 ± 2.3	45.5 ± 3.1

*Values are mean ± SD, n = 11*.

$\overset{\cdot }{\text{V}}$O_2_, oxygen uptake;

*, significant differences (p < 0.05) between pre and post confinement;

(†), *significant differences (p < 0.05) between post HYPOXIA and post NORMOXIA confinement*.

The hemoglobin level at the end of the HYPOXIA confinement was greater than that observed upon completion of the NORMOXIA confinement (Table 1).

As observed in Table 1, there was no significant change in the resting oxygen uptake ($\overset{\cdot }{\text{V}}$O_2_rest) and minute ventilation (${\overset{\cdot }{\text{V}}}_{\text{E}}$rest) during the 10-d NORMOXIA and HYPOXIA confinements. $\overset{\cdot }{\text{V}}$O_2_rest and ${\overset{\cdot }{\text{V}}}_{\text{E}}$rest were significantly higher on day 10 of the HYPOXIA confinement compared to the NORMOXIA confinment. $\overset{\cdot }{\text{V}}$O_2peak_ was not significantly affected by the NORMOXIA or HYPOXIA confinements (Table 1). These data have been discussed in a previous report (see Kounalakis et al., 2013).

In addition, no significant differences were observed in systolic arterial pressure (SAP) or diastolic arterial pressure (DAP), between before and after the NORMOXIA (SAP: pre = 118 ± 8 mmHg, post = 115 ± 8 mmHg; DAP: pre = 79 ± 6 mmHg, post = 75 ± 7 mmHg) and HYPOXIA (SAP: pre = 119 ± 9 mmHg, post = 123 ± 9 mmHg; DAP: pre = 80 ± 6 mmHg, post = 85 ± 9 mmHg).

**Figure 2:**
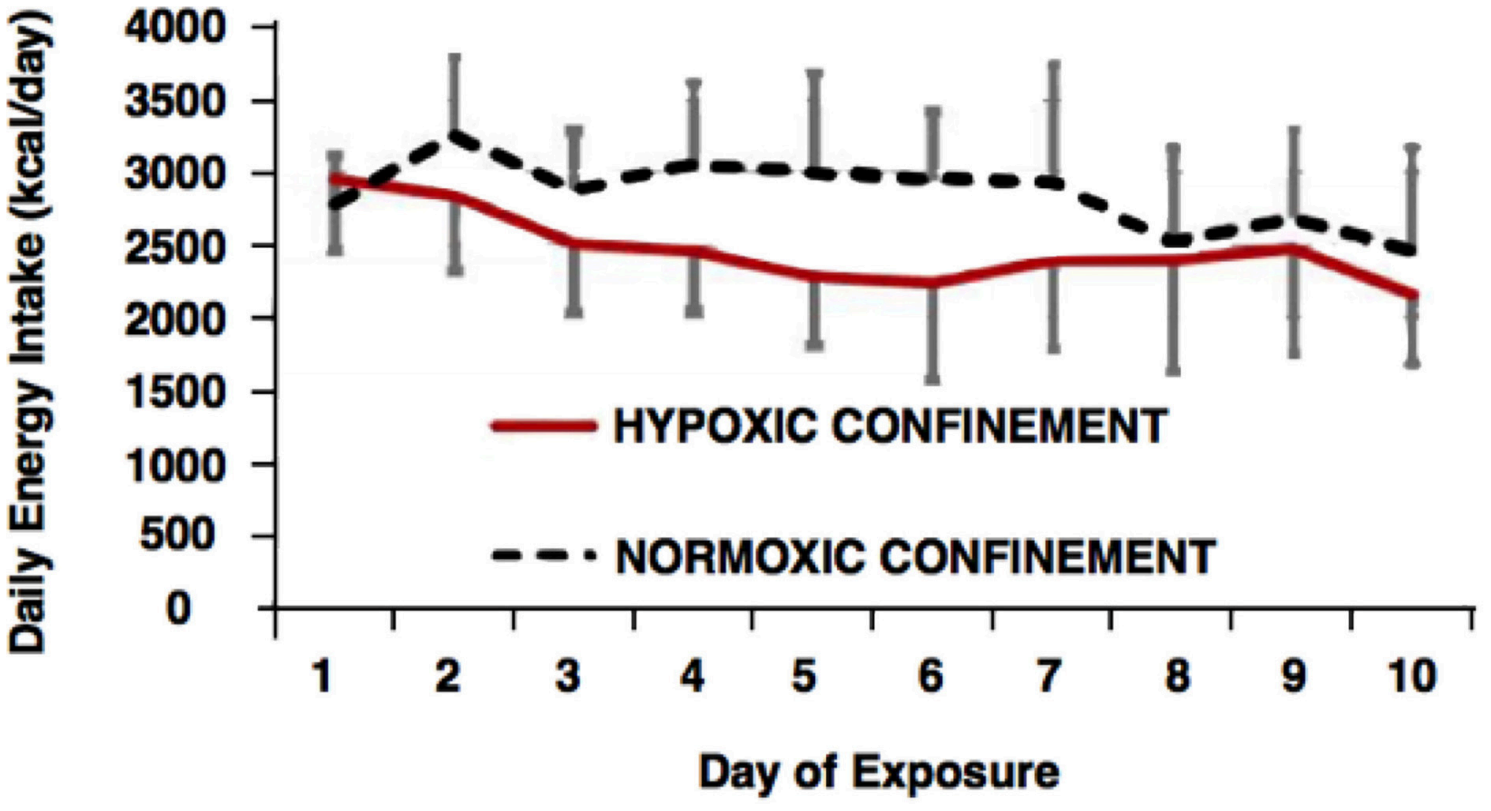
**Daily energy intake (kcal.day^**−1**^) during the normoxic and hypoxic confinements**.

A lower energy intake was observed in the HYPOXIA (*p* = 0.02), with mean daily energy intake being 2847 ± 241 and 2472 ± 251 kcal·day^−1^ in NORMOXIA and HYPOXIA, respectively (Figure 2).

The postprandial blood flow in the SMA increased after the meal, with peak values being observed 30 min postprandially (mean increase of 456 ± 15 ml·min^−1^) (Table 2). Thereafter, mesenteric arterial flow decreased, but remained elevated above the baseline value (by 123 ± 6 ml·min^−1^) for the remainder of the postprandial period. Mesenteric arterial flow response was not significantly different after the 10-day NORMOXIA and HYPOXIA confinements (Table 2). The coefficient of variation for the ultrasound measurements were 1.56% for arterial diameter, and 9.29% for arterial flow. The former can be attributed to the sonographer, and the latter to the physiological variability in the response.

**Table 2:** **Fasted and mean postraprandial mesenteric arterial blood flow, ghrelin, peptide YY_**3-36**_ (PYY), adrenaline and noradrenaline and subjective composite satiety score (CSS) evaluation before (PRE) and after (POST) the 10-day NORMOXIA and HYPOXIA confinements**.

		**Fasted state**	**Postprandial state**			
**Confinement**		**Time (min)**				
		**0**	**30**	**60**	**90**	**120**
**MESENTERIC ARTERIAL BLOOD FLOW (ml·min^−1^)**						
NORMOXIA	Pre	149.7 ± 6.7	530.6 ± 26.1	400.6 ± 20.7	279.0 ± 13.9	263.6 ± 14.7
	Post	131.8 ± 8.5	593.1 ± 20.8	409.5 ± 14.7	316.5 ± 13.7	251.7 ± 10.3
HYPOXIA	Pre	146.0 ± 8.0	627.1 ± 29.4	455.9 ± 22.8	287.0 ± 13.8	233.6 ± 10.5
	Post	142.8 ± 5.4	646.0 ± 19.1	420.5 ± 9.6	332.4 ± 13.2	316.2 ± 12.2
**GHRELIN (pg·ml^−1^)**						
NORMOXIA	Pre	1190.4 ± 34.2	1057.4 ± 22.2	959.9 ± 22.8	971.5 ± 22.8	962.1 ± 24.3
	Post	1163.3 ± 35.2	1010.7 ± 26.8	950.2 ± 23.4	842.9 ± 25.7	835.6 ± 24.6
HYPOXIA	Pre	1030.4 ± 32.4	921.2 ± 19.2	864.6 ± 19.6	830.1 ± 23.5	830.2 ± 19.4
	Post	1176.8 ± 32.4	1015.0 ± 24.0	935.6 ± 23.1	911.4 ± 21.9	919.6 ± 31.1
**LEPTIN (ng·ml^−1^)**						
NORMOXIA	Pre	3.02 ± 0.19				
	Post	3.56 ± 0.22				
HYPOXIA	Pre	2.60 ± 0.21				
	Post	3.63 ± 0.26				
**PYY (pg·ml^−1^)**						
NORMOXIA	Pre	100.0 ± 37.5	128.6 ± 31.1	118.1 ± 23.2	111.5 ± 21.0	131.9 ± 22.7
	Post	114.9 ± 28.8	144.6 ± 28.7	126.1 ± 24.6	129.5 ± 21.6	128.2 ± 24.3
HYPOXIA	Pre	97.7 ± 27.2	128.8 ± 41.5	118.0 ± 35.6	125.8 ± 33.7	122.1 ± 33.7
	Post	107.2 ± 19.4	142.8 ± 30.1	133.9 ± 31.4	137.1 ± 30.9	139.9 ± 31.1
**ADRENALINE (nmol·l^−1^)**						
NORMOXIA	Pre	0.31 ± 0.01	0.19 ± 0.01	0.21 ± 0.01	0.22 ± 0.01	0.26 ± 0.01
	Post	0.27 ± 0.01	0.15 ± 0.01	0.21 ± 0.01	0.21 ± 0.01	0.22 ± 0.01
HYPOXIA	Pre	0.30 ± 0.02	0.20 ± 0.02	0.18 ± 0.00	0.16 ± 0.01	0.24 ± 0.01
	Post	0.26 ± 0.01	0.16 ± 0.01	0.18 ± 0.00	0.17 ± 0.01	0.22 ± 0.01
**NORADRENALINE (nmol·l^−1^)**						
NORMOXIA	Pre	0.89 ± 0.02	1.02 ± 0.03	0.99 ± 0.03	0.98 ± 0.02	0.94 ± 0.03
	Post	0.77 ± 0.02	0.91 ± 0.02	0.81 ± 0.02	0.84 ± 0.03	0.86 ± 0.03
HYPOXIA	Pre	1.01 ± 0.03	1.19 ± 0.04	1.01 ± 0.03	0.99 ± 0.02	1.03 ± 0.03
	Post	1.30 ± 0.04^*^	1.52 ± 0.07	1.49 ± 0.05	1.52 ± 0.06	1.47 ± 0.05
**CSS EVALUATION (mm)**						
NORMOXIA	Pre	31.8 ± 2.0	53.1 ± 2.9	53.8 ± 3.4	41.8 ± 2.8	34.9 ± 2.8
	Post	31.1 ± 2.1	47.2 ± 2.7	44.4 ± 2.7	40.8 ± 2.5	38.0 ± 2.6
HYPOXIA	Pre	38.3 ± 2.2	55.9 ± 2.9	53.9 ± 2.8	48.0 ± 2.4	46.0 ± 2.6
	Post	37.3 ± 2.0	55.2 ± 2.9	55.3 ± 2.9	45.9 ± 2.8	44.6 ± 2.8

Values are mean ± SEM, n = 11.

CSS, composite satiety score;

*, significant differences between pre and post confinement; p < 0.05.

There were no changes in fasting ghrelin levels after each confinement (Table 2). Ghrelin decreased postprandially (*p* < 0.001), but the response did not change during any confinement, or differ between them (Table 2).

In the fasted state, PYY was not significantly different between the confinements. Postprandially, PYY increased (*p* < 0.001), in a similar manner (*p* = 0.46), in both confinements (Table 2).

In the fasted state, noradrenaline, but not adrenaline, was significantly increased only by HYPOXIA (*p* = 0.04). There was a statistical tendency for elevated postprandial levels of noradrenaline in the HYPOXIA (*p* = 0.07). There were no significant postprandial changes of adrenaline either before or after each confinement (Table 2).

Subjective satiety (CSS) in the fasted state remained unaltered after both confinements (*p* = 0.19). CSS increased after the test meal, but there were no differences between the confinements (Table 2).

Leptin levels in the fasted state significantly increased after both confinements. In NORMOXIA, the absolute leptin values in the fasting state increased from 3.0 ± 2.1 μg·l^−1^ to 3.6 ± 2.4 μg·l^−1^ (*p* = 0.04); while in HYPOXIA they increased from 2.6 ± 2.3 μg·l^−1^ to 3.6 ± 2.8 μg·l^−1^ (*p* = 0.02). There was no significant difference between the responses in NORMOXIA and HYPOXIA (*p* = 0.53) both with and without the change in fat mass used as a co-variate (*p* = 0.52).

**Figure 3:**
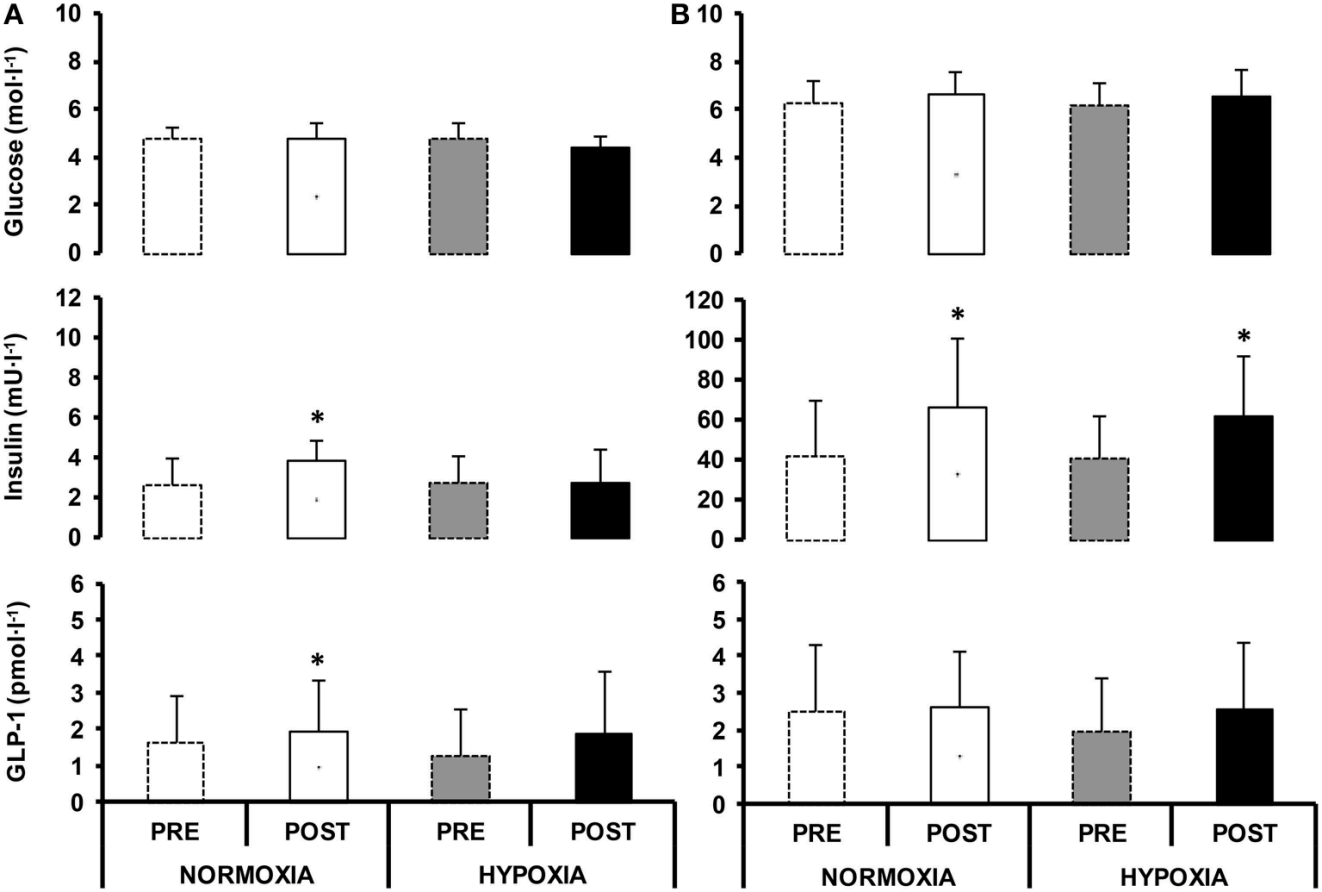
**Circulating glucose, serum insulin and GLP-1 values in fasted (A) and postprandial state (mean of 2 h, B), before (PRE) and after (POST) 10-day normoxic (NORMOXIA; A) and hypoxic (HYPOXIA; B) confinement**. Values are mean ± SEM. ^*^Indicates significant difference between PRE and POST confinement (*p* < 0.05).

**Figure 4:**
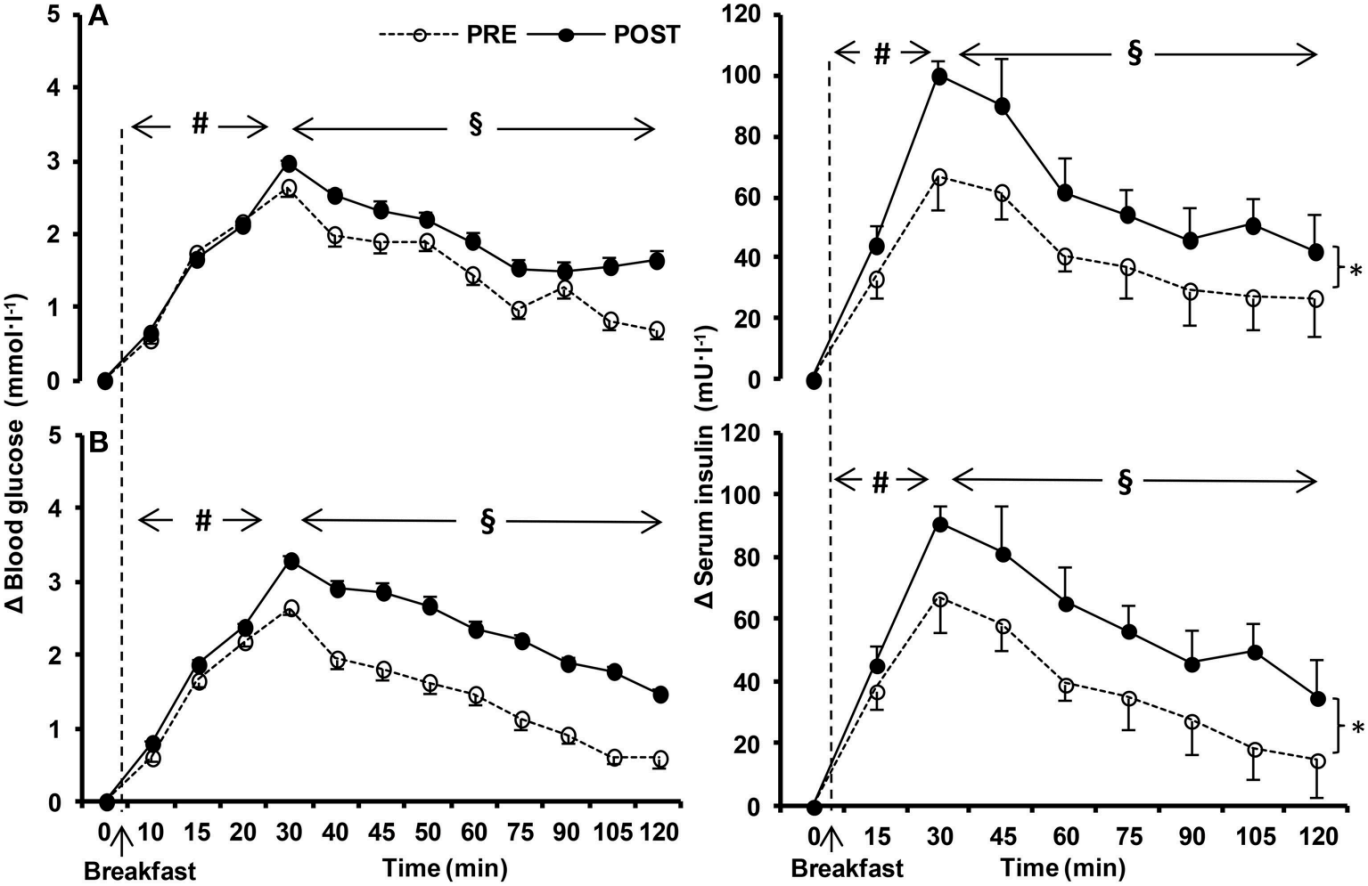
**Relative change (Δ) in postprandial circulating glucose, and serum insulin values before (PRE) and after (POST) the 10-day normoxic (NORMOXIC; A) and hypoxic (HYPOXIC; B) confinement**. Values are mean ± SEM. ^*^Indicates significant (*p* < 0.05) difference between PRE and POST confinement; #indicates significant (*p* < 0.05) differences over time from the fasted state; § indicates significant (*p* < 0.05) differences over time from the peak values.

Blood glucose levels in the fasted state were unchanged in NORMOXIA and HYPOXIA (Figure 3). Fasting insulin and fasting GLP-1 were higher after NORMOXIA (*p* = 0.01; *p* = 0.02), but not after HYPOXIA. Postprandially, glucose was elevated in NORMOXIA and HYPOXIA until the 40th min, and then gradually decreased (Figure 4). Postprandially, insulin initially increased in all conditions, and then gradually decreased until the end of the 2-h measurement. After the 10-day confinement period, insulin was higher (*p* < 0.05) in both NORMOXIA and HYPOXIA compared with the values observed in the pre-tests (Figure 4). GLP-1 increased after the meal, and the response was similar for both confinements (Figure 3).

HOMA IR significantly increased in NORMOXIA (pre: 0.6 ± 0.3, post: 0.9 ± 0.4; *p* = 0.03), while no differences were observed in HYPOXIA (pre: 0.5 ± 0.3, post: 0.5 ± 0.4). HOMA-β was significantly different after each confinement (*p* = 0.03), with no differences between them (NORMOXIA: pre: 46.9 ± 33.8, post: 81.5 ± 45.3 and HYPOXIA: pre: 57.7 ± 52.0, post: 64.5 ± 44.2; *p* = 0.77).

**Figure 5:**
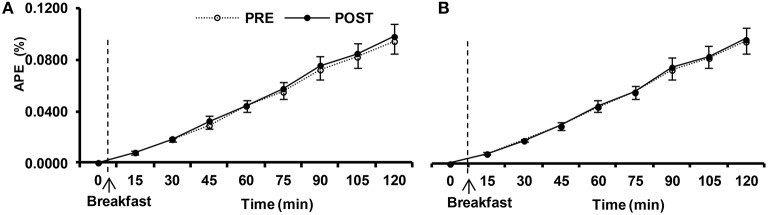
**Isotopic enrichement of expired CO_**2**_ expressed in atom percent excess (APE) in postprandial response before (PRE) and after (POST) the 10-day normoxic (NORMOXIC; A) and hypoxic (HYPOXIC; B) confinements**. Values are mean ± SEM. ^*^Indicates significant (*p* < 0.05) difference between PRE and POST confinement.

The enrichment of ^13^-C in expired air, derived from labeled glucose, increased after the meal (Figure 5; *p* < 0.001), although it did not differ between confinements (*p* = 0.71). Thus, there was no evidence of impaired dietary glucose availability following HYPOXIA, and the speed with which ingested glucose was absorbed and metabolized was not altered by this confinement.

## Discussion

The principal finding of the present study is that a continuous 10-day exposure to normobaric hypoxia without the confounding factor of strenuous physical activity caused a decrease in dietary intake. The higher resting oxygen uptake ($\overset{\cdot }{\text{V}}$O_2_rest) observed at the end of the hypoxic confinement compared to the normoxic confinement although statistically significant, may not be of physiological relevance. Namely, it may be argued that the observed significant differences in $\overset{\cdot }{\text{V}}$O_2_rest and ${\overset{\cdot }{\text{V}}}_{\text{E}}$rest observed in the present study are attributable more to the observed tendency, albeit not significant, of a decrease in $\overset{\cdot }{\text{V}}$O_2_rest and ${\overset{\cdot }{\text{V}}}_{\text{E}}$rest in the normoxic condition, rather than an increase in the hypoxia condition. We also observed a slight increase in body fat mass during NORMOXIA, whereas no change was observed during the HYPOXIA confinement.

Our observation of reduced energy intake during the HYPOXIA confinement concurs with the reported loss of appetite after a 31-day hypobaric hypoxic exposure in laboratory conditions (Westerterp-Plantenga et al., 1999), and the suppressed energy intake after a 7-h exposure to normobaric hypoxia (Wasse et al., 2012). The present subjects ate *ad libitum*, and no change in appetite was detected by the CSS after the test meal. A reduction in energy intake is consistent with the suggested altitude anorexia mediated by leptin (Tschöp et al., 1998), which is thought to regulate food intake and energy expenditure. In NORMOXIA the increased fasting leptin could be explained on the basis of the tendency for increased body fat mass, whereas in the HYPOXIA confinement the leptin increased, despite a trend for a reduced total fat content. This increase in leptin levels might be related to the decreased food intake. Our observed leptin response is in agreement with studies reporting increased leptin levels after hypoxic exposure (Shukla et al., 2005; Mazzeo, 2008; Snyder et al., 2008). Furthermore, it has been reported that hypoxia-treated cells up-regulate obese (ob) gene transcription, suggesting that enhancement of leptin secretion *in vivo* under hypoxic conditions may be a mechanism to consider when developing therapeutic methods for obesity treatment (Yingzhong et al., 2006). However, equivocal leptin levels after HYPOXIA, could be due to high intra-individual responses, as well as differences in the degree of hypoxic exposure (Mazzeo, 2008). In obesity leptin levels are already elevated but this does not lead to a suppression of energy intake so a further increase may not be of particular benefit.

Similar to other studies, we observed no change in ghrelin levels during the HYPOXIA confinement, although the ghrelin level decreased after the meal as expected in a normal postprandial response (Cummings et al., 2001). In agreement with others (Lippl et al., 2010), the HYPOXIA confinement did not significantly decrease the fasting ghrelin, most likely due to the fact that the absolute amount of weight loss was small. Unchanged ghrelin levels have also been reported, concomitant with significant reductions of 5 kg in body mass (Benso et al., 2007). It has been suggested that PYY can inhibit ghrelin neurons and consequently ghrelin secretion (Riediger et al., 2004). In the present study, PYY was unchanged after HYPOXIA, which does not support the theory that it contributes to high-altitude anorexia.

Westerterp et al. (1992, 1994), who observed reduced body weight mainly due to fat loss during a Mt. Everest climb (>5000 m, 3 weeks), reported no significant differences in resting metabolic rate measured by respiratory gas analysis at sea level and at high altitude. In most of the field studies, a number of environmental factors (hypobaric hypoxia, cold) and intense physical activity might affect metabolic rate. However, there are indications of increased REE in altitude studies (Grover, 1963; Gill and Pugh, 1964; Stock et al., 1978). Furthermore, Pulfrey and Jones (1996) reported that climbing to nearly 8000 m required 536 kcal/day just for the acclimatization to altitude, which did not include energy expenditure for physical exertion. Moreover, Butterfield et al. (1992) reported a 27% transient increase of REE on day 2 at altitude, followed by a plateau at 17% above the sea level REE by day 10. Reynolds et al. (1999) reported that REE ranged from 1.85 up to 3.82 times higher than the REE measured at sea level before the expedition, with the mean of 2.98 ± 0.70 for the climbers, and of 2.43 ± 0.45 for the base camp individuals. Again, all the previous evidence of increased REE at high altitude were related to even lower oxygen availability due to pressure change at high altitude. Lippl et al. (2010) confirmed that hypobaric hypoxia (with no physical activity) contributed to increased REE, but there was no evidence, that daily normobaric hypoxic confinement (frequently used as hypoxic training for athletes) could affect metabolic rate.

Circulating catecholamines can also stimulate EE (Cori and Buchwald, 1930). Our finding of increased noradrenaline after HYPOXIA is in line with other studies (Calbet, 2003; Richalet et al., 2010), indicating enhanced activation of the sympathetic nervous system (SNS) by hypoxia (Cutler et al., 2004; Gilmartin et al., 2008). It has been demonstrated that variability in REE can be explained by differences in SNS activity (Webber and Macdonald, 2000). The increase in REE induced by hypoxia *per se* may not be desirable for athletes training in hypoxic conditions, as their energy requirements may be higher. By contrast, this response to hypoxia might be beneficial for obese individuals, in whom exercise limitation can profoundly restrict daily activities, and lead to lower energy expenditure.

The weight loss at altitude may not be entirely due to an imbalance between the amount of food (energy) ingested and EE. Slowed, and possibly impaired, absorption of nutrients at altitude has also been suggested (Schoots et al., 2006) to reduce the availability of the ingested energy. In contrast, it has been reported that, at least up to an altitude of 5000 m, malabsorption does not seem to play a critical role in altitude-related weight loss (Kayser, 1994). In the present study, HYPOXIA did not compromise the gut blood flow response to the test meal. We did not observe any significant differences from the normal, anticipated increase after the meal (Sidery and Macdonald, 1994; Totman et al., 2009), nor were there differences in the responses of superior mesenteric arterial flow between the NORMOXIA and HYPOXIA confinements. Recently, it was suggested that the suppression of acylated ghrelin during 7 h of hypoxic exposure might be linked to impaired gut blood flow in response to acute hypoxia (Wasse et al., 2012). In addition, the observation of impaired postprandial gut blood flow (SMA) during acute altitude exposure as a consequence of increased intestinal sympathetic tone, suggests that, if sustained, may be related to reduced energy intake, during prolonged exposure (Loshbaugh et al., 2006). However, our results concur with the previously reported loss of appetite, which was not associated with any reductions in gut blood flow during several days of exposure to hypobaric hypoxia (Kalson et al., 2010). Thus, it appears unlikely that impaired gut blood flow could be responsible for high-altitude anorexia, as previously proposed. In addition, there were no changes in the postprandial ^13^CO_2_ level in expired air (derived from the ingested ^13^C-glucose) after confinement in NORMOXIA or in HYPOXIA. This, together with the finding of unaltered gut blood flow, provides evidence that HYPOXIA did not induce malabsorption, and thus did not reduce the availability of ingested energy.

Fasted insulin and GLP-1 were increased after the NORMOXIA, but not after the HYPOXIA confinement. Interestingly, a trend for statistical significance (*p* = 0.09) toward decreased fasting blood glucose after the HYPOXIA confinement was noted. Consequently, the HOMA IR index was elevated after the NORMOXIA confinement only, and the HOMA-β index was elevated after both the NORMOXIA and HYPOXIA confinements, due to increased fasting insulin. Post-prandially, a trend for elevated blood glucose only after the HYPOXIA confinement was concomitant with the elevated insulin response, which might suggest increased insulin resistance due to hypoxia or to the imposed inactivity (Hamburg et al., 2007; Olsen et al., 2008) in the physically well-trained participants. Moreover, the reduction in insulin-mediated peripheral glucose uptake could also be compounded by the small decrease of fat-free mass, and presumably also by the increase in SNS activation in hypoxia. Recently, Peltonen et al. (2012) demonstrated that SNS activation was also associated with the disruption of glucose control. Therefore, the observed tendency for insulin resistance could be, in part, due to a hypoxia-induced SNS activation. Stock et al. (1978) reported no changes either in fasting blood glucose levels, or in the postprandial blood glucose response during the 1st week of exposure to altitude (3650 m), while after the 2nd week at high altitude, there was a reduction in fasting and postprandial blood glucose, and insulin levels. In contrast to our study, the subjects in that study were exercising. In addition, compared to our study the technique of blood sampling was different; in particular, blood samples were drawn from the antecubital vein in the field, whereas in the present study arterialised venous blood samples were obtained. The former would lead to an underestimation of the glucose response to a meal due to tissue extraction. The observed tendency for an elevated postprandial blood glucose response observed in the HYPOXIA confinement was concomitant with unaltered adrenaline levels, in accordance with previous observations (Pulfrey and Jones, 1996). Moreover, our findings of significantly higher noradrenaline levels in the fasted state in hypoxia, with a tendency of elevated postprandial noradrenaline levels only after the HYPOXIA confinement are consistent with the observations of increased activity of the SNS, when oxygen availability is decreased (Gilmartin et al., 2008; Peltonen et al., 2012).

The observed reduction of the *ad libitum* energy intake in the HYPOXIA compared to the NORMOXIA confinement, most likely contributed to the loss of mass in the former condition. This is in line with the results of Abete et al. (2010), who reported a weight loss of 0.5–1.0 kg week^−1^ in subjects whose energy intake was restricted by 500–1000 kcal·day^−1^ compared to the usual diet.

## Methodological considerations

A study investigating the effect of a stressor/factor on metabolism, appetite and body composition, such as the present study, can be conducted using two principal methodological approaches. Either the subjects' energy intake is limited, but appropriately matching their energy expenditure, or the energy intake is not limited (*ad libitum*). In the present study we incorporated the latter approach, and thus did not anticipate any differences in satiety scores between the normoxic and hypoxic conditions. Differences would most likely have been evident with a limited energy protocol. We opted for the *ad libitum* protocol, since one of the principal aims of the study was to assess whether indications of hypoxic anorexia as experienced by alpinists was evident at lower altitude. Hypoxic anorexia has been reported during high altitude expeditions, and would not be of any significance at low altitudes, as simulated in the present study. Nevertheless, we reasoned that this phenomenon most likely starts to develop at some threshold altitude, and indications of its onset might be observed. The reduced energy intake observed in the hypoxia trial certainly suggests that this component of the hypoxic anorexia phenomenon is evident at altitudes simulated in the present study. Certainly, it would be worthwhile repeating this protocol at higher altitudes.

In both trials subjects were ambulatory, but did not participate in any physical exercise. The activity of the subjects was not monitored continuously during the 10-d confinements, but was similar. Any significant differences in physical activity would have contributed to the outcomes.

The conventional method for estimating resting energy expenditure is the ventilated hood method. This method is acceptable for environments in which the oxygen fraction is normal (FO_2_ = 0.2093) and does not fluctuate. In conditions, where the oxygen fraction has been reduced, such as in the present study, commercially available devices do not measure the oxygen fraction of the gas entering and exiting the hood. Only in this manner can the REE be reliably determined. In the present method we measured resting oxygen uptake just prior to a incremental load exercise. The subjects were resting, while seated on a cycle ergometer, and the resting oxygen uptake was measured over a 5-min period. Also, the subjects conducted the exercise ~4 h after consuming breakfast, and 1 h after a light snack. As a consequence the resting oxygen uptake values are higher than would be expected for a fasted individual resting supine for several hours.

The purpose of the NORMOXIA confinement trial in the present study was to be able to account for unexpected changes that would be observed due to confinement, such as the observed tendency for decreased resting oxygen uptake. Thus, if, for reasons unknown, confinement *per se* does tend to reduce oxygen uptake then our results also suggest that hypoxia is capable of counteracting such a decrease.

## Conclusions

In contrast to other studies, particularly field studies, we have minimized and/or eliminated certain confounding factors, which may contribute to weight loss during longer exposures at higher altitudes, including cold stress, strenuous physical activity, detraining, and unpalatable food. By carefully monitoring nutritional intake and minimizing physical activity of the subjects, our results focus on the direct effects of normobaric hypoxia in the fasted state on postprandial metabolic responses. Our results indicate metabolic changes, which may lead to reduction in body weight, but it remains speculative whether hypoxia of greater magnitude might induce more pronounced weight loss. In conclusion, athletes and alpinists conducting hypoxic training should be aware that hypoxia may lead to a negative energy balance as a consequence of reduced energy intake, and should monitor their energy intake to prevent such loss in mass, which may ultimately have a detrimental effect on performance.

## Author contributions

The project was conceived by IBM, OE, and IAM, who also designed the experimental protocol. All authors participated in the experiments. MA and LS analyzed the blood samples, RK was responsible for the ultrasonic measurement of mesenteric blood flow. SK and MK analyzed the oxygen uptake measurements. MA, SK, and IBM conducted the statistical analysis. IBM, MA, and IAM prepared the manuscript, which was then revised by all remaining authors.

### Conflict of interest statement

The authors declare that the research was conducted in the absence of any commercial or financial relationships that could be construed as a potential conflict of interest.
